# An evaluation of inflammatory gene polymorphisms in sibships discordant for premature coronary artery disease: the GRACE-IMMUNE study

**DOI:** 10.1186/1741-7015-8-5

**Published:** 2010-01-13

**Authors:** Benjamin D Brown, Jérémie Nsengimana, Jennifer H Barrett, Richard A Lawrence, Lori Steiner, Suzanne Cheng, D Timothy Bishop, Nilesh J Samani, Stephen G Ball, Anthony J Balmforth, Alistair S Hall

**Affiliations:** 1 Leeds Institute of Genetics, Health and Therapeutics (LIGHT), University of Leeds, UK; 2 Leeds Institute of Molecular Medicine (LIMM), University of Leeds, UK; 3 Roche Molecular Systems, Pleasanton, California, USA; 4 Department of Cardiovascular Sciences, University of Leicester, UK

## Abstract

**Background:**

Inflammatory cytokines play a crucial role in coronary artery disease (CAD). We investigated the association between 48 coding and three non-coding single nucleotide polymorphisms (SNPs) from 35 inflammatory genes and the development of CAD, using a large discordant sibship collection (2699 individuals in 891 families).

**Methods:**

Family-based association tests (FBAT) and conditional logistic regression (CLR) were applied to single SNPs and haplotypes and, in CLR, traditional risk factors of CAD were adjusted for.

**Results:**

An association was observed between CAD and a common three-locus haplotype in the interleukin one (*IL-1*) cluster with *P *= 0.006 in all CAD cases, *P *= 0.01 in myocardial infarction (MI) cases and *P *= 0.0002 in young onset CAD cases (<50 years). The estimated odds ratio (OR) per copy of this haplotype is 1.21 (95% confidence interval [95CI] = 1.04 - 1.40) for CAD; 1.30 (95CI = 1.09 - 1.56) for MI and 1.50 (95CI = 1.22 - 1.86) for young onset CAD. When sex, smoking, hypertension and hypercholesterolaemia were adjusted for, the haplotype effect remained nominally significant (*P *= 0.05) in young onset CAD cases, more so (*P *= 0.002) when hypercholesterolaemia was excluded. As many as 82% of individuals affected by CAD had hypercholesterolaemia compared to only 29% of those unaffected, making the two phenotypes difficult to separate.

**Conclusion:**

Despite the multiple hypotheses tested, the robustness of family design to population confoundings and the consistency with previous findings increase the likelihood of true association. Further investigation using larger data sets is needed in order for this to be confirmed.

See the related commentary by Keavney: http://www.biomedcentral.com/1741-7015/8/6

## Background

Coronary atherosclerosis is predominantly an asymptomatic process that progresses over the course of a lifetime. Arterial inflammation is central to plaque progression and plaque rupture with atherosclerotic lesions established as active sites of inflammation [1]. In particular, cytokines appear to coordinate the development of atherosclerosis leading to the formation of complex atherosclerotic plaques. These, in turn, can trigger acute thromboembolic complications such as myocardial infarction (MI) and the rupture/repair process that promotes the progression of luminal narrowing [2]. Furthermore, those lesions conveying the greatest risk to the individual are characterized by a higher level of inflammation. Coronary atherosclerosis and its complications are a complex disorder resulting from the combined effects of multiple environmental and genetic factors. Whilst most traditional risk factors for coronary artery disease (CAD) are themselves heritable, a family history remains an independent predictor of this condition [1,2]. The aim of this study was to evaluate individual polymorphisms and haplotypes in inflammatory genes for association with an increased predisposition to CAD. In total, 51 single nucleotide polymorphisms (SNPs) were analysed in 35 inflammatory genes in a large cohort of discordant sibships recruited across the UK in the Genetic Risk of Acute Coronary Events (GRACE) study.

## Methods

### Clinical methods

The study was approved by the Multicentre (MREC) and Local Research Ethics Committees (LRECs) throughout the UK. The details of the recruitment process can be found in the Additional File 1. Premature CAD was defined as a validated MI, percutaneous transluminal coronary angioplasty (PTCA), coronary artery bypass surgery (CABG) or angina (exercise test positive or angiogram showing at least one lesion >50%) before the age of 66. Hypercholesterolaemia was considered as either being on lipid lowering therapy or having total cholesterol greater than 4.9 mmol.l^-1 ^for individuals with a previous MI or greater than 5.2 mmol/l for individuals with other affected phenotypes (PTCA, CABG or angina) and unaffected individuals. Hypertension was either being on therapy or a blood pressure greater than 150/90 mmHg.

### Genotyping

Fifty SNPs and one insertion/deletion, mainly located in coding regions of 35 inflammatory genes, were genotyped (48 polymorphisms from coding regions and three from the introns). The primer mix and all other necessary components for the SNP genotyping were provided by Roche Molecular Systems (Pleasanton, USA). Characteristics of the polymorphisms are shown in the Additional File 2. DNA extraction and storage was performed using the PUREGENE^® ^DNA extraction kit (available from Gentra Systems, MN, USA). A pooled polymerase chain reaction (PCR) amplified the chosen targets from 50 ng of genomic DNA to produce biotin labelled products. These products were then hybridized with sequence specific oligonucleotide probes immobilized in a linear array. A series of development stages enabled detection of hybridization between probe and PCR product thus allowing identification of genotypes (homozygous wild, homozygous mutant, heterozygote). The processed strip was then scanned and genotypes were assigned by StripScan software (Roche Molecular Systems) [3] providing a semi-automated reading of the data assigning genotypes.

### Statistical analyses

Family relationships were validated using the Graphical Representation of Relationships (GRR) and RELATIVE programs [4,5] based on the 51 polymorphisms from this study plus 65 additional SNPs (analysis details in the Additional File 3). Allele frequencies were estimated using the program MENDEL which accounts for family relationships [6] and Hardy-Weinberg equilibrium was tested for each SNP using a goodness-of-fit test. The primary analysis was a test of association in presence of linkage between CAD and each individual SNP using the family-based association test (FBAT) [7]. We used the version of this test that employs empirical variance to account for residual familial correlation [8] (program FBAT 2.0.2C, option -e). An additive mode of inheritance was assumed. The analysis was repeated in two subsets of the data: (1) all cases affected with MI and their siblings and (2) all cases of CAD before the age of 50 and their siblings (see details in the Additional File 3). For genes or regions where two or more polymorphisms were in linkage disequilibrium (LD), haplotype analysis was also carried out in FBAT.

The haplotypes showing the highest evidence of association were further analysed using simple conditional logistic regression (CLR) in STATA [9], subsequently adding in clinical covariates. Robust variance was used to account for correlation between family members in CLR [10,11]. Haplotypes were inferred using the program HAPLORE [12]. As parents were not genotyped, there was some uncertainty in haplotype assignment. This uncertainty was accounted for in CLR by using a weighting approach originally designed for unrelated cases and controls [13] and adapted here to family data (see details in the Additional File 3). After identifying a haplotype associated with CAD, we used unconditional logistic regression to assess whether the same haplotype predicts hypercholesterolaemia, the most predominant clinical risk factor for CAD in our dataset.

## Results

### Family structures and effects of clinical risk factors

The initial data set consisted of 2870 individuals from 930 families. As a result of relationship checking, a total of 41 families were discarded - mainly because reported siblings were found to be more likely half-siblings. In addition, two families were each split into two, leaving a final sample size of 2699 persons from 891 families. The structure of the families included in this study is detailed in Table 1. The median age of disease onset was 50 years (range 21-66) and 69% of affected individuals had a MI as the index event. The median age of unaffected individuals was 57 years (range 35-85) and intra-family comparisons of ages revealed that over 80% of unaffected siblings had surpassed the age at which their affected siblings had their events. Clinical characteristics of the study sample are shown in Table 2.

**Table 1:** Structure of families included in the study.

No. of affecteds	No. of unaffecteds	No. of families with		
		
		CAD	CAD before 50 years*	MI †
1	1	164	149	122

1	2	439	235	362

1	>2	49	38	39

2	1	158	46	78

2	2	42	15	23

2	>2	18	6	14

≥ 3	≥1	21	4	11

Total families		891	493	660

Total affecteds		1154	568	802

Total unaffecteds		1545	855	1204

* In this setting, individuals affected after 50 years were considered as unphenotyped

† For this trait coding, individuals free of myocardial infarction (MI) but affected by other forms of coronary artery disease (CAD) were considered unphenotyped.

**Table 2:** Clinical characteristics of the study sample.

	Unaffected	Affected		
			
Risk factor	*N *(%)	*N *(%)	Univariate *P*-value *	Multivariable OR†
Total sample	1545 (100)	1154 (100)	-	-

Male sex	630 (40.8)	873 (75.7)	<0.0001	4.71 (3.65, 6.08)

Diabetes mellitus	62 (4.0)	117 (10.1)	<0.0001	1.63 (1.00, 2.66)

Hypertension	391 (25.3)	522 (45.2)	<0.0001	1.99 (1.52, 2.60)

Hypercholesterolaemia	454 (29.4)	945 (81.9)	<0.0001	10.70 (8.11, 14.11)

Ever smoked	856 (55.4)	849 (73.6)	<0.0001	1.72 (1.32, 2.25)

BMI	Mean (SD) 27.16 (4.62)	Mean (SD) 27.74 (4.05)	0.0002	1.00 (0.97, 1.04)

* Univariate conditional logistic regression for binary factors and linear regression clustered by family for body mass index (BMI)

† Odds ratio (OR) and 95% confidence intervals from multivariable conditional logistic regression analysis including all six factors

SD: standard deviation

Considered individually, each of the traditional risk factors of CAD [male gender, diabetes, hypertension, hypercholesterolaemia, cigarette smoking and body mass index (BMI)] has a highly significant effect on disease risk. When all these factors are included in the model jointly, male gender, hypertension, cigarette smoking and hypercholesterolaemia still show a highly significant effect on CAD, whilst diabetes mellitus has a borderline effect and BMI was not associated with CAD (Table 2).

### Family-based association tests for individual SNPs

The results of SNP by SNP analysis are shown in Table 3. Results are reported only for SNPs that were in Hardy-Weinberg equilibrium and for which we had at least 50 informative families (the average number of informative families was 345). Associations with CAD significant at 5% level were found for polymorphisms in interleukin 9 [(*IL9*) C4244T, *P *= 0.01], nitric oxide synthase 3 [(*NOS3*) A498G, *P *= 0.05], complement component 5 [(*C5*) A2416G, *P *= 0.04], interleukin 4 receptor [(*IL4R *T1682C, *P *= 0.01 and A1902G, *P *= 0.04] and eotaxin [(*SCYA11*) G361A, *P *= 0.04]. In the subsets of MI cases and those with younger CAD onset (<50 years) the associations with *C5 *(A2416G) remained (MI only *P *= 0.006; <50 years *P *= 0.04) and additional significant associations were observed with interleukin 1 alpha (*IL1α*) C549T (MI only *P *= 0.02; <50 years *P *= 0.02).

**Table 3:** Tests of association between SNPs and CAD using FBAT.

Chromosome	Gene	SNP	RS number	MAF	*P*-value *
1p32-p31	*VCAM1*	T707C	1041163	0.16	0.39

1q21-24	*SELP*	G40A	6131	0.18	0.84
		
		G75271T	6133	0.14	0.67

1q22-q25	*SELE*	A153C	5361	0.11	0.33

1q31-32	*IL10*	C8700A	1800872	0.24	0.29

2q12-q21	*IL1α*	C549T	1800587	0.31	0.08

2q14	*IL1β*	C4336T	1143634	0.26	0.58
		
		C1423T	16944	0.34	0.49

2q33	*CTLA4*	C875T	5742909	0.10	0.34

2q33	*CTLA4*	A1241G	231775	0.40	0.68

3p21	*CCR2*	G46295A	1799864	0.08	0.88

3p21	*CCR5*	Wt/del 580-611	333	0.14	0.72
		
		G59029A	1799987	0.44	0.33

3p21.3	*CCR3*	C320T	5742906	0.01	- †

3p26-p24	*IL5RA*	G482A	2290608	0.28	0.10

4q12-q13	*GC*	G35706T	7041	0.44	0.50
	
	*GC*	C35717A	4588	0.29	0.21

5q22-32	*CD14*	C2232T	2569190	0.47	0.63

5q23-q31	*IL4*	C582T	2243250	0.14	0.66

5q31	*IL13*	C4045T	1295686	0.20	0.78

5q31	*TCF7*	C883A	5742913	0.12	0.35

5q31.1	*TCF7*	A383T	25882	0.14	0.49

5q31.1	*CSF2*	T2600C	244656	0.22	0.16

5q31-q32	*ADRB2*	A1633G	1042713	0.37	0.91
		
		C1666G	1042714	0.42	0.37
		
		C2078T	1800888	0.02	- †

5q31-q35	*IL9*	C4244T	2069885	0.13	**0.01 (T)**

5q35	*LTC4S*	A620C	730012	0.31	0.22

6p21.3	*LTA*	A1069G	909253	0.39	0.60
	
	*TNF*	G3787A	1800629	0.21	0.34
		
		G3857A	361525	0.07	0.99

7p21-p15	*IL6*	G589C	1800796	0.06	0.33
		
		G987C	1800795	0.43	0.38

7q35-q36	*NOS3*	A498G	1800779	0.40	**0.05 (G)**
		
		G7002T	1799983	0.36	0.67

9q32-q34	*C*5	A2416G	17611	0.44	**0.04 (G)**

10q11.1	*SDF1*	G880A	1801157	0.20	0.84

11q11-qter	*UGB*	G587A	3741240	0.36	0.65

11q13	*FCERB1*	A7297G	569108	0.03	0.48

12q13.1	*VDR*	T12022C	2228570	0.40	0.77
		
		G45082A	1544410	0.42	0.61

16p11.2-p12.1	*IL4R*	A398G	1805010	0.46	0.56
		
		T1682C	1805015	0.20	**0.01 (C)**
		
		A1902G	1801275	0.25	**0.04 (G)**

17q11.2-q12	*NOS2A*	C231T	1137933	0.23	0.98

17q21.1-q21.2	*SCYA11*	G361A	3744508	0.19	**0.04 (G)**
		
		G1169A	4795895	0.20	0.47

19q13.1	*TGFB1*	C629T	1800469	0.27	- ‡

19p13.2	*ICAM1*	A120T	5491	0.001	- ‡
		
		G657A	1799969	0.12	0.43

19p13.3-p13.2	*C*3	C364G	2230199	0.24	0.39

* In parenthesis after the *P*-value: the allele associated with an increased disease risk.

† Number of informative families below 50.

‡ Not in Hardy-Weinberg equilibrium.

MAF, minor allele frequency; SNP, single nucleotide polymorphism; CAD, coronary artery disease.

### Haplotype analysis

More than one SNP were genotyped within 13 genes and one gene cluster (*IL1α-IL1β*). Table 4 shows the genes or gene cluster where any haplotype was significantly associated with CAD, CAD before 50 years or MI at the nominal 5% level using FBAT. The LD (measured with D') between adjacent SNPs is also shown along with their physical position from dbSNP build 128. The most significantly associated haplotype was found in the *IL1 *cluster (CCC haplotype, frequency of 41% in the total sample), which was found at increased frequency in all CAD cases (*P *= 0.006), MI cases (*P *= 0.01) and young CAD cases (*P *= 0.0002), although the overall haplotype effect only reached the 5% significance level in early-onset CAD cases (*P *= 0.02, with 7 degrees of freedom). In *NOS3 *gene, there was a significant protective effect of the AT haplotype (frequency 11%) in overall CAD (single haplotype *P *= 0.009 and overall test *P *= 0.04) and in MI (single haplotype *P *= 0.005, overall test *P *= 0.03). However, there was no evidence of haplotype association in this gene in younger disease onset. Other haplotypes with nominally significant effects were found in *IL4R *and *SCYA11 *(Table 4).

**Table 4:** Haplotype association to CAD, CAD before age 50 (CAD50) or MI.

Gene	SNP	Position *	LD†	Hap^‡^	Freq^§^	P_CAD	P_MI	P_CAD50
IL1α	C549T	113259431		CCC	0.41	**0.006 (+)**	**0.01 (+)**	**0.0002 (+)**
				
			0.78	CCT	0.26	0.60	0.96	0.32
				
*IL1β*	C4336T	113306861		CTC	0.17	0.30	0.06	0.31
				
			0.67	TCC	0.06	0.08	0.30	0.08
				
*IL1β*	C1423T	113311338		TCT	0.04	0.45	-	0.37
				
				CTC	0.03	0.69	-	-
				
				TTT	0.02	-	-	-
				
				CTT	0.01	-	-	-
				
				All (7df)	-	0.13	0.25	**0.02**

*NOS3*	A498G	150320876		AG	0.50	0.77	0.84	0.63
				
			0.47	GT	0.23	0.24	0.32	0.40
				
*NOS3*	G7002T	150327044		GG	0.16	0.16	0.11	0.36
				
				AT	0.11	**0.009 (-)**	**0.005 (-)**	0.15
				
				All (3df)	-	**0.04**	**0.03**	0.39

*SCYA11*	G361A	29637007		GG	0.66	**0.05 **(+)	0.10	**0.02 (+)**
				
			1.00	GA	0.18	0.84	0.73	0.29
				
*SCYA11*	G1169A	29635559		AG	0.16	**0.02 **(-)	0.09	0.06
				
				AA	0.00	-	-	-
				
				All (2df)	-	**0.05**	0.17	0.06

*IL4R*	A398G	27263704		ATA	0.45	0.16	0.36	0.15
				
			0.37	GTA	0.33	0.44	0.46	0.63
				
*IL4R*	T1682C	27281681		GCG	0.11	**0.04 **(+)	0.24	0.56
				
			1.00	ACG	0.06	0.22	0.49	0.20
				
*IL4R*	A1902G	27281901		ATG	0.03	0.91	0.60	**0.03 (+)**
				
				GTG	0.02	-	-	-
				
				GCA	0.002	-	-	-
				
				ACA	0.001	-	-	-
				
				All (7df)	-	0.88	0.41	0.17

*Physical position (base pairs) from NCBI dbSNP build 128.

† Absolute value of the linkage disequilibrium (LD) coefficient D' between consecutive single nucleotide polymorphisms (SNPs).

‡ Results only shown for haplotypes having a number of informative families >50.

§Haplotype frequencies estimated in the total sample.

+ (-), Haplotype associated with increased (decreased) disease risk; CAD, coronary artery disease; MI, myocardial infarction; SNP, single nucleotide polymorphism.

### IL1 haplotype effect estimation

Using an additive model with no covariate adjustment in weighted CLR, each copy of *IL1*-CCC haplotype confers an increase in risk of CAD with estimated odds ratio (OR) of 1.21 [95% confidence interval (95 CI) = 1.04-1.40, *P *= 0.01] in the total sample. For young-onset cases, the estimated OR was 1.50 (95 CI = 1.22 - 1.86, *P *= 0.0001) and for MI the OR was 1.30 (95 CI = 1.09 - 1.56, *P *= 0.003). The overall significance level of all haplotypes (7df) was 0.28 in the whole sample, 0.18 for MI only and 0.02 in the affecteds before age 50. When tested jointly with four clinical covariates (hypercholesterolaemia, hypertension, smoking and sex), the effect of haplotype *IL1*-CCC on CAD was no longer significant in the whole sample (OR = 1.07, *P *= 0.57) nor in those affected by MI (OR = 1.26, *P *= 0.07), but it was nominally significant in younger cases (<50 years OR = 1.39, *P *= 0.05). No significant interaction was found between *IL1*-CCC haplotype and any of the covariates.

The most potent predictor of increased risk of disease in our population was hypercholesterolaemia (see Table 2). When it was excluded from the multivariable analysis, the effect of haplotype *IL1*-CCC in the whole sample was significant (OR = 1.22, *P *= 0.05), and was more so in those with younger onset disease (OR = 1.53, *P *= 0.002, see Table 5). This indicates a possible relationship between the haplotype and hypercholesterolaemia itself. In order to test this, a simple unconditional logistic regression analysis was carried out on the whole study population and stratifying by CAD, using hypercholesterolaemia as the trait of interest with *IL1*-CCC haplotype as predictor. In the whole dataset, the estimated OR of having hypercholesterolaemia was 1.15 (95 CI = 1.03 - 1.29, unadjusted *P *= 0.01) per copy of haplotype *IL1*-CCC. This was comparable to the OR of developing CAD per copy the same haplotype using unconditional logistic regression (OR = 1.21, 95 CI = 1.02 - 1.43, unadjusted *P *= 0.02). The haplotype effect on hypercholesterolaemia was of the same magnitude in subjects affected and unaffected by CAD, although not significant in these smaller subsets (OR = 1.10, 95 CI = 0.88-1.37 and OR = 1.13, 95 CI = 0.96-1.33, respectively). This result suggests that the haplotype *IL1*-CCC may increase the risk of CAD through increasing the risk of hypercholesterolaemia, although the high correlation between hypercholesterolaemia and CAD in our study makes this difficult to evaluate.

**Table 5:** Joint effects of the *IL1*-CCC haplotype and other risk factors on CAD.

Trait	Factor	*P*_value	Odds ratio	95 CI*	
CAD	*IL1 *CCC	**0.047**	1.22	1.00	1.47
	
	Sex (baseline = female)	<0.001	4.86	4.00	5.91
	
	Ever smoked	<0.001	2.01	1.62	2.50
	
	Hypertension	<0.001	3.16	2.55	3.92
	
	Overall test (4df)	*P *< 0.0001			

CAD before 50 years	*IL1 *CCC	**0.002**	1.53	1.17	2.01
	
	Sex (baseline = female)	<0.001	6.44	4.78	8.67
	
	Ever smoked	<0.001	3.03	2.16	4.26
	
	Hypertension	<0.001	3.57	2.56	5.00
	
	Overall test (4df)	*P *< 0.0001			

* 95% confidence interval (CI)

CAD, coronary artery disease.

## Discussion

We present here the results of an association analysis of CAD with multiple inflammatory genes in one of the UK's largest discordant sibship studies. This family-based cohort avoids the potential bias of population stratification and admixture that may affect even a well designed case-control study [14]. The haplotype analysis maximized the use of family information and accommodated phase uncertainty. Our strongest finding was a suggested association with a common haplotype in the interleukin 1 gene cluster (*IL1*-CCC, see Table 4), particularly in those individuals with younger onset CAD. The per-copy OR for CAD was 1.21 (unadjusted *P *= 0.01), rising to 1.50 in younger affecteds (unadjusted single haplotype test *P *= 0.0001 and overall test *P *= 0.02). The effect of this haplotype on early-onset disease risk remained significant after adjusting for covariates other than hypercholesterolaemia (OR = 1.53, *P *= 0.002, Table 5). However, including hypercholesterolaemia in the model greatly reduced the estimated effect. The effect of *IL1*-CCC on hypercholesterolaemia was found to be of similar magnitude as on CAD itself, suggesting that *IL1*-CCC may actually increase the risk to CAD through increasing the risk of hypercholesterolaemia.

The relationship between hypercholesterolaemia and CAD is well established. At present, no direct evidence of correlation between *IL-1 *and hypercholesterolaemia exists in man. Mice lacking interleukin-1 receptor antagonist (*IL1RA*) display significant derangement of cholesterol homeostasis in response to an atherogenic diet [15]. Furthermore, studies in hypercholesterolemic mice suggest that lack of *IL-1β *or over-expression of *IL1RA *can partly protect against atherosclerosis [16]. Accordingly, elevation of *IL-1 *is consistently observed in individuals with unstable CAD and predicts mortality post MI [17]. The use of HMG-CoA inhibitors (statins), known to reduce lipoprotein levels and risk of cardiovascular events, has been shown to reduce mRNA levels of both *IL1α *and *IL1β *in peripheral blood mononuclear cells (PBMCs) [17] providing further evidence of an interplay between *IL-1 *and hypercholesterolaemia.

Ikonomidis *et al*. [18] have evaluated the immediate and short-term effects of anakinra, a recombinant IL1 receptor antagonist, on coronary flow; left ventricular (LV) and endothelial function and mediators of inflammation. In patients with rheumatoid arthritis and no evidence of CAD/ischaemia, anakinra resulted in both an acute and sustained improvement in left ventricular function, endothelial function and coronary flow reserve with a reduction in IL-6 and endothelin-1, compared to placebo/prednisolone. Relatively small numbers of patients were studied and those with CAD were specifically excluded. It remains unclear, therefore, whether these findings have any relevance to those with CAD/without rheumatoid arthritis. In particular, are the changes in LV and endothelial function reversible when ischaemic in aetiology and are the inflamed joints of active rheumatoid arthritis a prerequisite for anakinra to have an effect? In animal models, acute anakinra administration does appear to reduce cardiomyocyte apoptosis and adverse remodelling [19]. Further clarity is likely to be added by the Medical Research Council funded ILA HEART study [20] evaluating anakinra in patients presenting with a MI.

The *IL1β *polymorphism C1423T has been investigated previously in relation to CAD, with mixed results. An excess of CC genotypes was reported in affected individuals compared to unaffecteds (22% versus 13%), although this difference was not significant [21]. Another study [22] subsequently found no evidence of difference in allele frequencies on this SNP between subjects with angiographically normal and abnormal coronary arteries. By contrast, Iacoviello *et al*. [23] found a significantly reduced risk of MI and ischaemic stroke at young age (<45 years for men and <50 for women) in carriers of the T allele after adjustment of the traditional risk factors. They also demonstrated that mononuclear cells in carriers of the T allele produce significantly lower *IL-1β *levels than in non-carriers. However, the same T allele was found significantly associated with increased risk of atherogenesis in subclavian arteries in a cohort of elderly Japanese [24].

In our study, no association was found between the *IL1β *C1423T SNP and CAD, but we did detect an association with a haplotype containing this SNP. These results suggest that the SNP itself may not increase the risk of CAD but may be in strong LD with a causal variant, which may also partially explain inconsistencies in results across different studies. It is also possible that it is the haplotype that influences CAD rather than one nucleotide change. Chen *et al*. [25] showed that SNP alleles in *IL1β *have increased transcriptional activity when combined (into haplotypes) and suggested that it may be a common feature of gene regulatory regions. *IL1 *gene cluster haplotypes have been found associated with many diseases, including schizophrenia and bipolar disorder [26], gastric cancer [27] and psoriatic arthritis [28]. In the case of CAD, age of onset appears to be an important factor (our study and [23]).

The *IL1α *and *IL1β *genes lie on chromosome 2 about 15 Mb centromeric to the linkage peak found in a large UK study of sibling pairs affected with CAD [29]. The evidence for linkage in this region was much stronger in the subset of sibling pairs without hypercholesterolaemia, although when this covariate was included in the analysis the main linkage peak shifted slightly further away from the *IL1 *gene cluster [30]. It is well known that the highest linkage peak may be at a considerable distance from the disease-causing locus, especially when based on relative pair analyses, so it is possible that the *IL1 *cluster contributed to the evidence of linkage.

Our second interesting finding was in the gene *NOS3 *on chromosome 7. Many studies have reported an association between the G7002T SNP in this gene (also referred to as 894G/T or Glu298Asp) and a number of diseases including CAD/MI [31,32] and essential hypertension [33,34]. However, most of these studies were based on unrelated case-control designs with a limited size. While our larger study does not confirm the association of this SNP with CAD (*P *= 0.67, Table 3), we did observe a weak association between CAD and a promoter region SNP (A498G, *P *= 0.05, Table 3) whilst a haplotype spanning the two SNPs showed a stronger association (single haplotype test *P *= 0.009 for CAD and *P *= 0.005 for MI, overall haplotype test *P *= 0.04 for CAD and *P *= 0.03 for MI, Table 4). The AT haplotype appears to reduce the risk of CAD and MI, although this was not confirmed in patients affected at younger age. Although the polymorphism G7002T changes the protein sequence (Glu298Asp), its actual functional significance is not well understood [33] and our results suggest again that a haplotype or an untyped SNP may be of relevance.

Other SNPs or haplotype associations marginally significant in this study were found in *IL9, C5, IL4R *and *SCYA11 *(Tables 3 and 4). Their significance was much lower than that of IL1 cluster and *NOS3*, and given the number of tests performed, they are more likely to be false positives. The *IL1 *cluster and *NOS3 *associations may also be due to type 1 errors and do not reach 5% statistical significance if adjusted for the number of tests performed. However, for the *IL1 *gene cluster, the consistency with prior findings [21,23,24,29,30] and the increased risk observed in younger subjects increase the likelihood that this is a true association, and the family-based design of our study provides reassurance against confounding by population stratification. Further investigation of a denser set of SNPs in this region in larger samples of patients and controls is needed for confirmation.

## Abbreviations

BMI: body mass index; CABG: coronary artery bypass surgery; CAD: coronary artery disease; CLR: conditional logistic regression; FBAT: family-based association test; LD: linkage disequilibrium; LV: left ventricular; MI: myocardial infarction; PCR: polymerase chain reaction; PTCA: percutaneous transluminal coronary angioplasty; SNP: single nucleotide polymorphism.

## Competing interests

The authors declare that they have no competing interests.

## Authors' contributions

The authors' contributions to this study were as follows: design/planning - ASH, NJS, SGB, DTB, AJB and JHB; study set-up - BDB and RAL; SNPs selection/assay production - SC and LS; lab supervision - AJB; statistical analyses - JN and JHB; paper drafting - BDB, JN and JHB; overall supervision - ASH. All the authors read and approved the manuscript.

## Pre-publication history

The pre-publication history for this paper can be accessed here:

http://www.biomedcentral.com/1741-7015/8/5/prepub

## Supplementary Material

Additional file 1 Details on the recruitment of study participants. Click here for file

Additional file 2 List of the polymorphisms analysed in this study (Table S1), with all their characteristics (chromosome region, long and short gene name, common SNP name, rs number, amino acid change). Click here for file

Additional file 3 Details on the statistical methods in data validation and genetic association testing. Click here for file
