# Supplementary material for: An evaluation of inflammatory gene polymorphisms in sibships discordant for premature coronary artery disease: the GRACE-IMMUNE study
Source: BMC Med. 2010 Jan 13;8:5. doi: 10.1186/1741-7015-8-5 (PMC2823655; doi:10.1186/1741-7015-8-5)
Supplement: Additional file 1 — Details on the recruitment of study participants. [file 1741-7015-8-5-S1.DOC]

# Additional file 1: Details on the recruitment process

Publicity for the GRACE study was generated through a National bus tour, media coverage (TV, radio, local newspaper) and posters in General Practitioner (GP) surgeries and hospitals. Interested individuals contacted our research nurses via freepost or free phone. Suitability for the study was assessed by the nurse and information sent to the initial contact person, both for the proband and his/her siblings. After a period of time for the family to consider the information the proband was contacted again, and if a sufficient number of siblings were willing to take part, contact details were taken and a research nurse obtained a detailed past medical and family history. The family history was obtained from one or more siblings, from whoever the family agreed had most knowledge. Validation of cardiac history was by means of a ‘yes/no’ GP questionnaire confirming the cardiac history reported by the patient. In the event of any discrepancies between the subject and GP information, the GP was contacted by phone and final arbitration performed by a cardiologist. Unaffected siblings were defined as having no clinical evidence of CAD, and medical histories were again validated by means of a GP questionnaire. In the final protocol, the patient information sheet and consent form had been approved by both Multicentre (MREC) and Local (LREC) Research Ethics Committees throughout the UK. Full approval was obtained before collection of either data or samples. Trust approval nationally via the Research and Development network was also obtained to allow phlebotomy at local hospitals as required.
