# Supplementary material for: An evaluation of inflammatory gene polymorphisms in sibships discordant for premature coronary artery disease: the GRACE-IMMUNE study
Source: BMC Med. 2010 Jan 13;8:5. doi: 10.1186/1741-7015-8-5 (PMC2823655; doi:10.1186/1741-7015-8-5)
Supplement: Additional file 2 — List of the polymorphisms analysed in this study (Table S1), with all their characteristics (chromosome region, long and short gene name, common SNP name, rs number, amino acid change). [file 1741-7015-8-5-S2.DOC]

**Table S1 (Additional file 2)**: Characteristics of genotyped polymorphisms

| **Chromosome region** | **Gene name** | **Acronym** | **SNP** | **RS number** | **AA (*)** |
| --- | --- | --- | --- | --- | --- |
| 1p32-p31 | Vascular cell adhesion molecule-1 | *VCAM1* | T707C | 1041163 | (-1594) |
| 1q21-24 | P-Selectin | *SELP* | G40A | 6131 | S330N |
|  |  |  | G75271T | 6133 | V640L |
| 1q22-q25 | E-Selectin | *SELE* | A153C | 5361 | S128R |
| 1q31-1q32 | Interleukin 10 | *IL10* | C8700A | 1800872 | (-571) |
| 2q12-q21 | Interleukin 1 alpha | *IL1α* | C549T | 1800587 | (-889) |
| 2q14 | Interleukin 1 beta | *IL1β* | C4336T | 1143634 | F105F |
|  |  |  | C1423T | 16944 | (-511) |
| 2q33 | Cytotoxic T lymphocyte antigen 4 | *CTLA4* | C875T | 5742909 | (-318) |
|  |  |  | A1241G | 231775 | T17A |
| 3p21 | Chemokine receptor 2 | *CCR2* | G46295A | 1799864 | V62I |
| 3p21 | Chemokine receptor 5 | *CCR5* | Wt/del 580-611 | 333 | del |
|  |  |  | G59029A | 1799987 | (-2454) |
| 3p21.3 | Chemokine receptor 3 | *CCR3* | C320T | 5742906 | P39L |
| 3p26-p24 | Interleukin 5 receptor antagonist | *IL5RA* | G482A | 2290608 | (-80) |
| 4q12-q13 | Group specific component | *GC* | G35706T | 7041 | E416D |
|  |  |  | C35717A | 4588 | T420K |

Table S1 continued

| **Chromosome region** | **Gene name** | **Acronym** | **SNP** | **RS number** | **AA (*)** |
| --- | --- | --- | --- | --- | --- |
| 5q22-32 | Cluster of differentiation 14 | *CD14* | C2232T | 2569190 | (-260) |
| 5q23-q31 | Interleukin-4 | *IL4* | C582T | 2243250 | (-590) |
| 5q31 | Interleukin-13 | *IL13* | C4045T | 1295686 | (intron 3) |
| 5q31 | Transcription Factor 7 | *TCF7* | C883A | 5742913 | P19T |
|  |  |  | A383T | 244656 | (-1459) |
| 5q31.1 | Colony stimulating factor 2 | *CSF2* | T2600C | 25882 | I117T |
| 5q31-q32 | Adrenergic beta-2 receptor | *ADRB2* | A1633G | 1042713 | R16G |
|  |  |  | C1666G | 1042714 | Q27E |
|  |  |  | C2078T | 1800888 | T164I |
| 5q31-q35 | Interleukin 9 | *IL9* | C4244T | 2069885 | T113M |
| 5q35 | Leukotriene C4 synthase | *LTC4S* | A620C | 730012 | (-444) |
| 6p21.3 | Lymphotoxin alpha | *LTA* | A1069G | 909253 | (intron A) |
|  | Tumour necrosis factor alpha | *TNF* | G3787A | 1800629 | (-308) |
|  |  |  | G3857A | 361525 | (-238) |
| 7p21-p15 | Interleukin 6 | *IL6* | G589C | 1800796 | (-572) |
|  |  |  | G987C | 1800795 | (-174) |
| 7q35-q36 | Nitiric oxide synthase 3 | *NOS3* | A498G | 1800779 | (-948) |
|  |  |  | G7002T | 1799983 | E298D |

Table S1 continued

| **Chromosome region** | **Gene name** | **Acronym** | **SNP** | **RS number** | **AA (*)** |
| --- | --- | --- | --- | --- | --- |
| 9q32-q34 | Complement component 5 | *C5* | A2416G | 17611 | I802V |
| 10q11.1 | Stromal derived factor 1 | *SDF1* | G880A | 1801157 | (+800) |
| 11q11-qter | Uteroglobin | *UGB* | G587A | 3741240 | (+38) |
| 11q13 | Beta subunit of the high-affinity IgE receptor | *FCERB1* | A7297G | 569108 | E237G |
| 12q13.1 | Vitamin D receptor | *VDR* | T12022C | 2228570 | M1T |
|  |  |  | G45082A | 1544410 | (intron 8) |
| 16p11.2-p12.1 | Interleukin-4 receptor | *IL4R* | A398G | 1805010 | I75V |
|  |  |  | T1682C | 1805015 | S503P |
|  |  |  | A1902G | 1801275 | Q576R |
| 17q11.2-q12 | Nitiric oxide synthase 2A | *NOS2A* | C231T | 1137933 | D346D |
| 17q21.1-q21.2 | Eotaxin | *SCYA11* | G361A | 3744508 | A23T |
|  |  |  | G1169A | 4795895 | (-1328) |
| 19p13.2 | Intercellular adhesion molecule-1 | *ICAM1* | A120T | 5491 | K56M |
|  |  |  | G657A | 1799969 | G241R |
| 19p13.3-p13.2 | Complement component 3 | *C3* | C364G | 2230199 | R102G |
| 19q13.1 | Transforming Growth Factor Beta 1 | *TGFB1* | C629T | 1800469 | (-509) |

(*) Amino-acid substitution or nucleotide number relative to transcription start site
