# Supplementary material for: An evaluation of inflammatory gene polymorphisms in sibships discordant for premature coronary artery disease: the GRACE-IMMUNE study
Source: BMC Med. 2010 Jan 13;8:5. doi: 10.1186/1741-7015-8-5 (PMC2823655; doi:10.1186/1741-7015-8-5)
Supplement: Additional file 3 — Details on the statistical methods in data validation and genetic association testing. [file 1741-7015-8-5-S3.DOC]

**Additional file 3: Details on statistical analyses**

## Data validation

Monozygotic twins were identified using GRR. Half-siblings (HS) and unrelated pairs were identified using the RELATIVE program as follows: for each reported full sibling (FS) pair, prior probabilities of 0.90 for FS status, 0.08 for HS and 0.02 for being unrelated were specified. Sibling pairs were confirmed as FS if their posterior probability of being FS, computed using RELATIVE, was greater than 0.90. If the posterior probability of being a FS pair was lower than 0.90, one of the subjects was excluded from the family. Posterior probabilities of relatedness were computed based on the 51 SNPs of Table S1 plus 65 additional polymorphisms.

## SNP by SNP and haplotype association analysis with FBAT

The principle of the FBAT method used in the primary analysis consists in comparing the observed genotype distribution in affected siblings to its expected distribution under the null hypothesis. With no parents genotyped, the expected distribution is estimated by considering all possible joint parental genotypes consistent with the offspring genotypes. The analysis was applied in the whole dataset and in two subsets of the data: considering all cases affected with MI and their siblings, and all cases of CAD before the age of 50 and their siblings. For these analyses, subjects affected by CAD but not by the sub-phenotype were treated as having unknown phenotype; in this way their genotypes contribute to the construction of parental genotypes but not to the contrast between affecteds and unaffecteds.

Haplotype analysis with FBAT was carried for genes or regions where two or more polymorphisms were in LD (measured with D’>0.30, estimated ignoring relatedness). In this analysis, the distribution of joint parental haplotypes is inferred from the offspring genotypes using the expectation-maximization algorithm, assuming no recombination between loci within families. The haplotype-based association test is therefore only applied to very closely linked loci. For SNPs having a rare allele, many families show no variation and are thus uninformative, and the analyses are restricted to SNPs/haplotypes with at least 50 informative families. Two types of haplotype tests were performed: the first test compares each haplotype to all others and gives an indication of the direction of the effect (increased/decreased risk) but suffers from the multiple-testing problem. The second test considers the joint effect of all the haplotypes in a region, which is less affected by multiple testing but lacks power when there are many haplotypes due to the resulting high number of degrees of freedom.

## Haplotype analysis with weighted CLR

Haplotypes were inferred using the program HAPLORE which, as parents were not genotyped, inferred all possible mating types consistent with the offspring genotypes, resulting in numerous possible haplotype assignments to each individual (i.e. multiple consistent family configurations). HAPLORE assigns a pair of haplotypes to each family member for each consistent family configuration and estimates the probability of this particular configuration. (This method of assigning haplotype probabilities ignored phenotype information, which may lead to a slightly conservative test and estimate of effect size if the alternative hypothesis is true).

Simple and multiple CLR analyses were clustered on family configuration and the probability of each configuration was used as a weight to allow for the uncertainty that is inherent in haplotype assignment. This approach of weighted CLR is built on a method originally designed for unrelated cases and controls and we adapted it to family data. As hypercholesterolemia was the predominant clinical risk factor and appeared to be correlated with the haplotype, logistic regression was applied with this factor as outcome. Unconditional logistic regression was used for this analysis to avoid losing a major part of the data as the design was not optimised to have discordant families for hypercholesterolemia.
